# Danshen injection ameliorates unilateral ureteral obstruction-induced renal fibrosis by inhibiting ferroptosis via activating SIRT1/GPX4 pathway

**DOI:** 10.3389/fphar.2024.1503628

**Published:** 2025-01-13

**Authors:** Yiwen Cao, Huan Zhao, Shuyin Lin, Junqi Chen, Jingli Xiong, Zhijun Zeng, Ziyu Long, Yingru Su, Yingqi Zhong, Lingru Zhao, Mingshan Zhang, Junbiao Wu, Yuan Zhou, Jiuyao Zhou

**Affiliations:** ^1^ Department of Pharmacology, School of Pharmaceutical Sciences, Guangzhou University of Chinese Medicine, Guangzhou, Guangdong, China; ^2^ The Fourth Clinical Medical College, Guangzhou University of Chinese Medicine, Shenzhen, Guangdong, China; ^3^ The Second Clinical Medical College, Guangzhou University of Chinese Medicine, Guangzhou, Guangdong, China

**Keywords:** danshen injection, renal fibrosis, ferroptosis, SIRT1, GPX4

## Abstract

**Introduction:**

The pathogenesis of renal fibrosis is related to blood stasis, and the method of promoting blood circulation and removing blood stasis is often used as the treatment principle. Danshen injection (DSI) is a commonly used drug for promoting blood circulation and removing blood stasis in clinic. However, whether DSI slows the progression of renal fibrosis or the potential mechanism is uncertain.

**Methods:**

We investigated renal fibrosis models using UUO mice and TGF-β stimulation in HK-2 cells.

**Results:**

Our findings revealed that DSI or Fer-1 alleviated kidney injury by ameliorating renal morphology injury and pathological injury *in vivo*. Besides, DSI or Fer-1 inhibited renal fibrosis *in vivo* and in TGF-β-induced HK-2 cells. Furthermore, ferroptosis was lessened under DSI or Fer-1 treatment. More importantly, the DSI active ingredients (danshensu, salvianolic acid B, protocatechuic aldehyde, caffeic acid and tanshinone IIA) could bind to SIRT1. The protein levels of SIRT1 and GPX4 were downregulated accompanied by the incremental concentrations of TGF-β or Erastin, which were repaired by DSI or Fer-1 intervention. However, the inhibition of ferroptosis and renal fibrosis owing to DSI were reversed by SIRT1 inhibitor EX527.

**Conclusion:**

Taken together, our results indicated that DSI could protect against ferroptosis to attenuate renal fibrosis by activating the SIRT1/GPX4 pathway. It is expected to be a potential agent to treat renal fibrosis.

## Graphical Abstract

**Figure:**
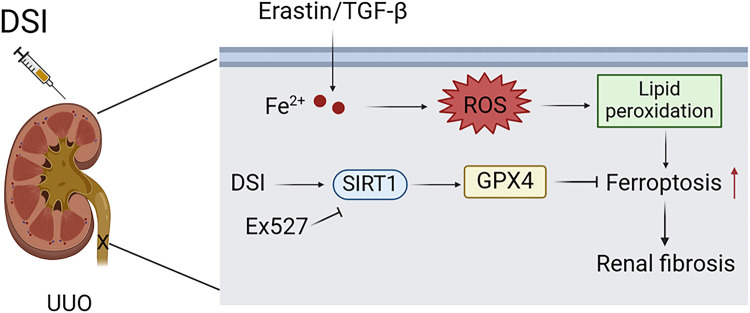
Mechanism diagram of DSI in UUO-induced mice. Danshen Injection inhibits ferroptosis via up-regulating SIRT1/GPX4 pathway to attenuate renal fibrosis.

## Introduction

Renal fibrosis, characterized by undue extracellular matrix (ECM) deposition, destroys and replaces the function of renal intrinsic cells, eventually leading to end-stage renal disease (ESRD) (Li et al., 2022a). Considered to be the ultimate outcome of various chronic kidney disease (CKD), renal fibrosis affects beyond 10% of the world population, and the available or affordable treatments are limited (Djudjaj and Boor, 2019). Currently, the strategies being developed for renal fibrosis mainly focus on enzymes closely related to ECM. Since these enzymes are usually multifunctional, treatments may trigger an immune response or other side effects (Li et al., 2022a). Due to limited effectiveness in treating renal fibrosis, there has been little progress in reversing fibrosis in the current decade, which has grown into the central issue of preclinical studies.

Ferroptosis, a recently identified metabolic cell death, features by iron overload and loss of antioxidant capacity, leading to the formation of lipid peroxidation products (Stockwell, 2022). During the process of renal fibrosis, disrupted kidney increased tubular iron filtration and excessive ingestion of exogenous iron. Renal parenchymal cells are further damaged by iron excess, which causes ferroptosis. Fibroblasts can then be activated or undergo direct EMT transformation into myofibroblasts, which exacerbates renal fibrosis (Zhang et al., 2022). Indeed, available evidence supports that targeting ferroptosis has therapeutic benefits in preclinical models of renal fibrosis (Sanz et al., 2023). Many drugs or compounds, such as Rosiglitazone, Linagliptin, Baicalein, Liproxstatin-1 and Ferrostatin-1 (Fer-1), have been reported to inhibit ferroptosis as well as renal fibrosis, which provides a new direction for us to find effective drugs without side effects.

Danshen, a prominent traditional Chinese medicine (TCM), is the dried root of the plant *Salvia miltiorrhiza* Bunge, which has been used to treat various diseases, including kidney diseases, cardiovascular and cerebrovascular diseases for many years (Hao et al., 2020; Wang et al., 2020). Danshen Injection (DSI) is an aqueous extract of Danshen and is widely used to treat CKD. Several researches have documented that DSI exerts anti-inflammatory, anti-oxidation and anti-fibrosis effects to protect renal function in CKD (Yin et al., 2014; Xu et al., 2016). Our previous research found that DSI could significantly induce autophagy to attenuate CKD via PI3K/AKT/mTOR pathway (Chen et al., 2022). However, the mechanism of how DSI improves fibrosis through inhibiting ferroptosis to curb the progression of CKD has not been completely investigated.

In this study, we purposed to elucidate the impact of DSI on ferroptosis as well as the underlying mechanism of DSI attenuating renal fibrosis.

## Materials and methods

### Reagents and antibodies

DSI was obtained from Chiatai qingchunbao Pharmaceutical Co., Ltd. (SFDA approval no. Z33020177, LOT no. 2,103,212, Zhejiang, China). Fer-1, Erastin and EX527 were obtained from Med Chem Express (Shanghai, China). Malonaldehyde (MDA) (A003-1-2 for kidneys, A003-4-1 for HK-2 cells), and glutathione (GSH) (A006-2–1) kits were obtained from Jiancheng Bioengineering Institute (Nanjing, China). Transforming growth factor-β (TGF-β) was obtained from PeproTech (Rocky Hill, NJ, United States). CCK-8 Assay Kit was gained from APExBIO Technology LLC (United States) and iron assay kit (E-BC-K772-M) was obtained from Elabscience (Wuhan, China). E-Cadherin (20,874–1-AP) was gained from Proteintech (Wuhan, China). Vimentin (5741S), α-smooth muscle actin (α-SMA) (19245S) and Sirtuin-1 (SIRT1) (9475S) were purchased from Cell Signaling Technology (Inc., United States). Glutathione peroxidase 4 (GPX4) (ab125066) and GAPDH (ab9485) were gained from Abcam (Cambridge, UK).

### Animal administration

Adult male C57BL/6J mice, weighing 17–20 g (special pathogen-free level, 7–8 weeks-old), were supplied by the Guangdong Medical Experimental Animal Center (Certificate Number: SCXK 2022–0002). The experimental protocols were strictly approved by the Animal Ethics Committee of Guangzhou University of Chinese Medicine (Ethics approval number: ZYD-2022–209).

Thirty-six C57BL/6J mice were assigned to the following six groups: sham group, unilateral ureteral obstruction (UUO) group, UUO + DSI-L group, UUO + DSI-M group, UUO + DSI-H group and UUO + Fer-1 group. After anesthetized with 1% sodium pentobarbital (50 mg/kg), the mice were placed on the operating table at a constant temperature of 37°C. The left proximal ureter of UUO mice was selected and ligated at two different sites using 4–0 silk sutures. The sham group only exposed the ureter.

**FIGURE 1:**
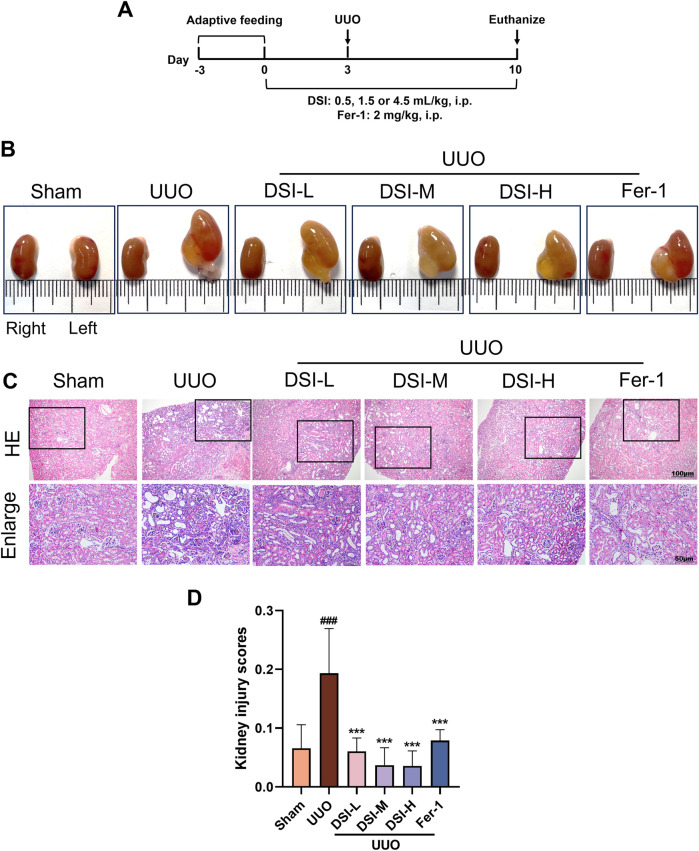
DSI alleviated renal pathological injury in UUO-induced mice. **(A)** Schematic diagram of the animal experimental design. **(B)** The representative morphology of kidneys. **(C, D)** Representative pictures and the kidney injury scores of HE staining (100 μm and 50 μm). Data were expressed as the mean ± SD (*n* = 6). ^*###*^ *p* < 0.001 vs Sham group, *p* < 0.001 vs UUO group.

Mice were pretreated with DSI (0.5, 1.5 and 4.5 mL/kg) and Fer-1 (2 mg/kg) respectively for 3 days before surgery and 7 consecutive days after surgery via intraperitoneal injection, while the other mice in the sham and UUO groups were given with the same volumes of 0.9% NaCl solution intraperitoneally (Figure 1A). After the experiment, the blood and kidneys of all groups were collected and stored at – 80°C for further study.

### Renal morphology analysis

All collected kidneys were placed on white paper with a ruler and photographed with a camera (Nikon, Tokyo, Japan) for morphological detection.

### Renal pathological analysis

Half of the kidneys were sliced vertically and soaked in 4% paraformaldehyde for 24 h. Then the samples were dehydrated with ethanol, hyalinized, immersed in wax and embedded in paraffin. Next, 5 μm-thick sample slides were stained with Hematoxylin and eosin (HE) or Masson’s Trichrome (Masson). Finally, the slides of each group were obtained under a light microscope (BX53, Olympus, Tokyo, Japan). Images of each group were randomly selected, and the area of renal tubular vacuoles and collagen deposition were quantitatively analyzed by ImageJ (v1.53) software. The ratio of positive area to total area in the visual field was selected as the renal injury score and fibrosis area.

### Cell culture and treatment

HK-2 cells were gained from Cell Resource Center in Institute of Biochemistry and Cell Biology (Procell Life Science & Technology Co.,.Ltd., China), and grown in DMEM complete media. When the growth density reached 70%–80%, HK-2 cells were incubated with TGF-β (10 ng/mL) + DSI (0.78, 1.56 and 3.12 μL/mL), Erastin (5 μM) + DSI (0.78, 1.56 and 3.12 μL/mL), Fer-1 (5 μM), TGF-β (10 ng/mL) + EX527 (10 μM), TGF-β (10 ng/mL) + EX527 (10 μM) + DSI (3.12 μL/mL) for 24 h, respectively.

### Cell viability analysis

5 × 10^3^ cells/well of HK-2 cells were seeded onto a plate. Subsequently, HK-2 cells were treated with DSI (0, 0.78, 1.56, 3.12, 6.24, 12.48 and 24.96 μL/mL), Erastin (0, 1, 2.5, 10, 20 and 40 μM), 10 ng/mL of TGF-β or 5 μM Erastin with or without DSI (0.78, 1.56 and 3.12 μL/mL) and 5 μM Fer-1 for 24 h, respectively. The cell viability was tested using CCK-8 Assay Kit.

### Detection of iron content, MDA and GSH levels

100 mg kidney tissues from each group were homogenized by homogenizer (JXFSTPRP-24L, Shanghai Jingxin industry development co., ltd.). *In vitro*, HK-2 cells were collected and homogenized by the ultrasonic cell disrupter (Diagenode, Belgium). The iron content, MDA and GSH levels of kidney tissues and HK-2 cells were determined using assay kits according the manufacturer’s instructions.

### ROS determination

The method of ROS detection was the same as what we described before (Cao et al., 2023). Briefly speaking, for UUO mice, frozen sections of kidney were incubated in 1 × clean working solution (Bestbio, BB-470516) at room temperature for 5 min. Then, the kidneys were incubated with DHE active oxygen fluorescence probe (1:1,000) for 30 min at 37°C. Washed with PBS for thrice, the samples were placed on a confocal laser microscope (LSM 800, ZEISS, Germany). Subsequently, the fluorescence intensity of ROS in each group was captured and counted by the ImageJ 1.48 software.

For cultured HK-2 cells, after being incubated with related reagents, the cells were stained with DCFH-DA (Beyotime, S0033S) at 37°C in the dark for 20 min. Next, the cells washed with cell culture medium thrice were collected and measured by a flow cytometer (FACSverse, BD Biosciences, United States).

### Molecular docking

The structures of active components of DSI (danshensu, salvianolic acid B, protocatechuic aldehyde, caffeic acid and tanshinone IIA) were downloaded from the PubChem database. Water molecules and original ligands were removed from the protein structures using AutoDock Tools (version 1.5.6). The structure of SIRT1 was downloaded from the Protein Data Bank (PDB) database (https://www.rcsb.org). AutoDock Vina (version 1.1.2) was used for SIRT1 and components docking. Finally, their docking modes were visualized by PyMOL.

### Western blot analysis

Protein levels of kidneys and HK-2 cells were measured by the BCA protein detection kit (Beyotime, Shanghai, China). Total proteins were separated by 10% SDS-PAGE gels and transferred onto a 0.22 μm PVDF membrane (Millipore, Bedford, MA, United States). The membranes blocked with 5% BSA for 2 h at room temperature were incubated with the antibodies against E-Cadherin, Vimentin, α-SMA, SIRT1, GPX4 and GAPDH at 4°C overnight. Then the proteins were incubated with secondary antibodies for 40 min at room temperature. Blots were measured by an enhanced chemiluminescence kit (Millipore, Bedford, MA, United States) and quantified by ImageJ 1.48 software.

### Statistical analysis

All data were shown as mean ± standard deviation (SD) and were statistically analyzed by SPSS 20.0 (IBM SPSS Statistics, Chicago, IL, United States). One-way analysis of variance (ANOVA) was used for groups of data. The differences were considered statistically significant when *p* < 0.05.

## Results

### DSI alleviated renal pathological damage in UUO-induced mice

To estimate the effect of DSI on UUO-induced mice, renal function indices were examined. Compared to the sham group, urine in the UUO group could not be excreted and remained in the kidney due to UUO operation, causing abnormal enlargement of the kidney. The abnormal morphological changes induced by UUO operation were markedly improved by DSI or Fer-1 (Figure 1B). The improvement of renal injury could be directly reflected by the morphology of the kidney to some extent. HE staining results revealed that, when compared to the UUO group, DSI or Fer-1 administration could decrease glomerular atrophy, tubular dilatation, brush boundary loss and inflammatory cell infiltrations (Figures 1C,D). In general, these results above indicated that DSI or Fer-1 could alleviate the morphological and pathological changes induced by UUO.

### DSI improved renal fibrosis in UUO-induced mice

**FIGURE 2:**
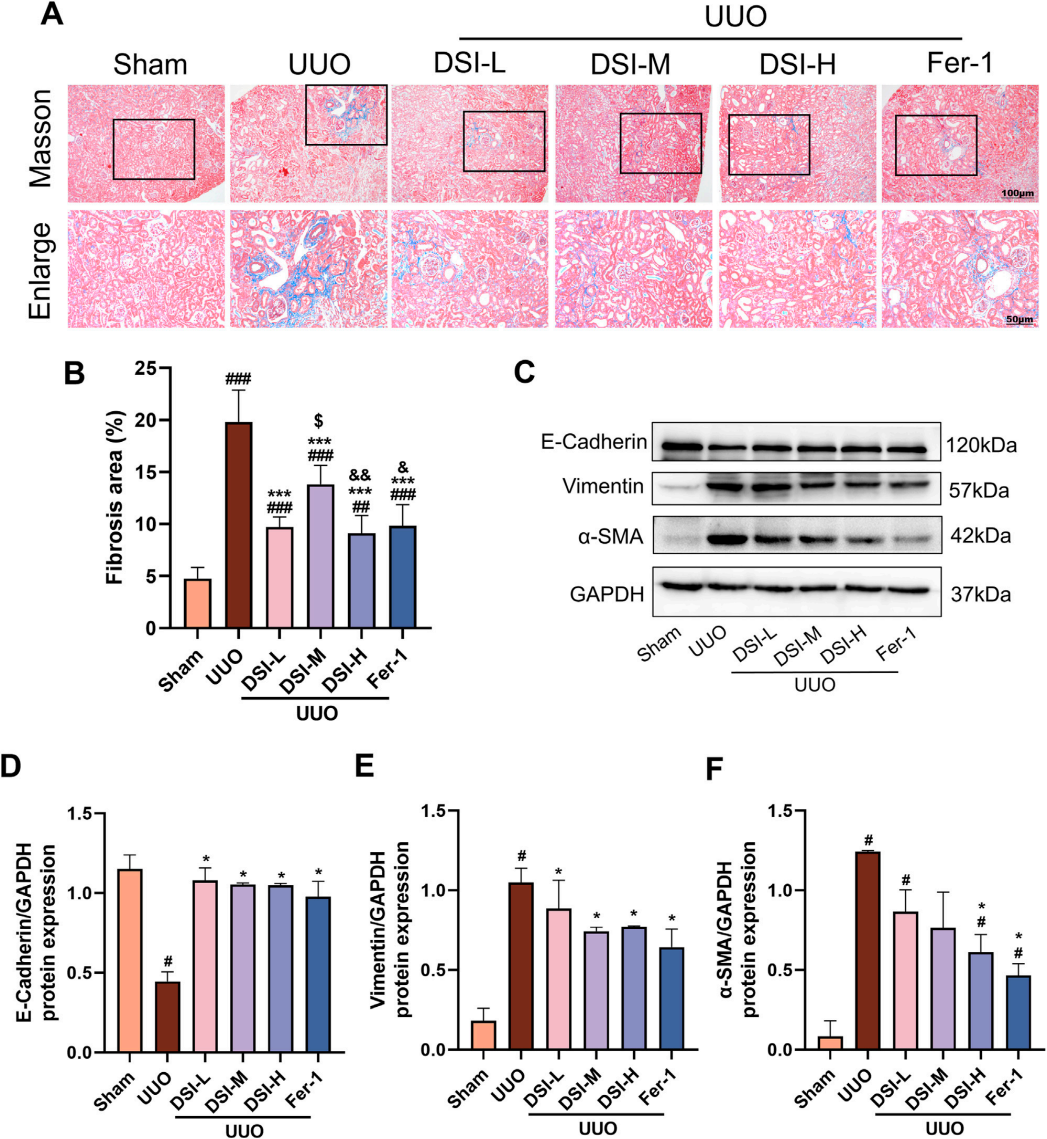
DSI improved renal fibrosis in UUO-induced mice. **(A, B)** Representative pictures and the fibrosis area of Masson staining (100 μm and 50 μm, *n* = 6). **(C–F)** Western blot analyses and quantitative results of E-Cadherin, Vimentin and α-SMA. Data were expressed as the mean ± SD (*n* = 3). ^*#*^ *p* < 0.05, ^*##*^ *p* < 0.01 and ^*###*^ *p* < 0.001 vs Sham group, ^*^ *p* < 0.05 and ^***^ *p* < 0.001 vs UUO group, ^*$*^ *p* < 0.05 vs DSI-L group, ^*&*^ *p* < 0.05 and ^*&&*^ *p* < 0.01 vs DSI-M group.

Abnormal collagen deposition is a characteristic of renal fibrosis (Buchtler et al., 2018). Masson staining results revealed that there is a marked increase in fibrotic area in the UUO group compared to the sham group, which was improved by DSI or Fer-1 treatment (Figures 2A,B). Epithelial-mesenchymal transition (EMT) is the predominant stage of renal fibrosis (Wang et al., 2023). Western blot results showed that the level of E-cadherin was significantly decreased, whereas Vimentin and α-SMA were significantly increased in the UUO group compared with that in the sham group. Intervention with DSI markedly ascended the level of E-cadherin and inhibited the upregulation of Vimentin and α-SMA (Figures 2C–F). Our data demonstrated that DSI or Fer-1 could effectively inhibit UUO-induced renal fibrosis.

### DSI inhibited TGF-β-induced fibrosis in HK-2 cells

**FIGURE 3:**
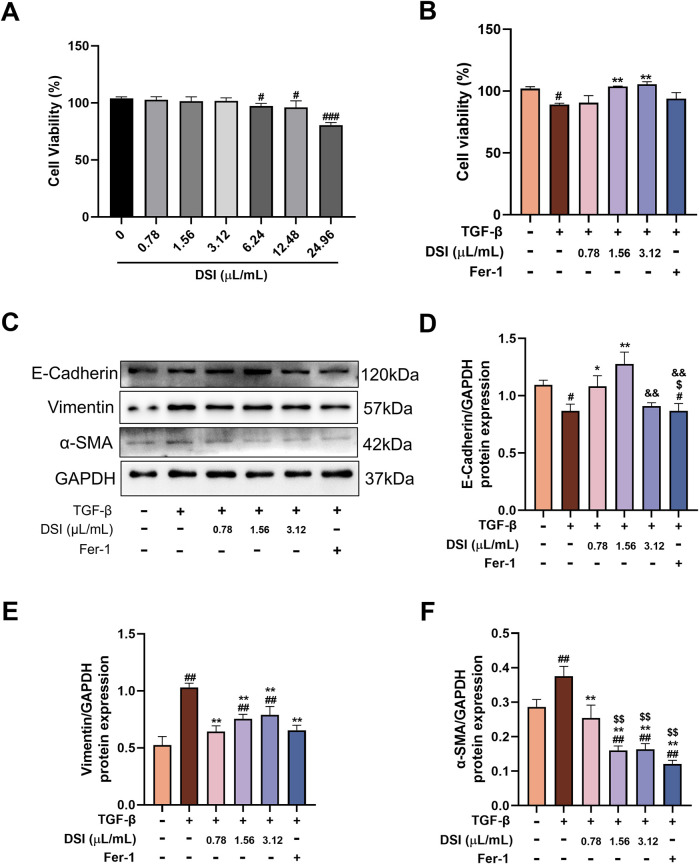
DSI inhibited TGF-β-induced fibrosis in HK-2 cells. **(A)** The cell viability of HK-2 cells tested by CCK8 Assay Kit. **(B)** The cell viability of HK-2 cells measured by CCK8 Assay Kit. **(C–F)** Western blot analyses and quantitative results of E-Cadherin, Vimentin and α-SMA. Data were expressed as the mean ± SD (*n* = 3). ^*#*^ *p* < 0.05 ^*##*^ *p* < 0.01 and ^*###*^ *p* < 0.001 vs Control group, ^**^ *p* < 0.01 and ^***^ *p* < 0.001 vs TGF-β group, ^*$*^ *p* < 0.05 and ^*$$*^ *p* < 0.01 vs 0.78 μL/mL DSI group, ^*&&*^ *p* < 0.01 vs 1.56 μL/mL DSI group.

Renal tubules are the majority constituent of the kidney and also the major positions to response to renal fibrosis (Zhang et al., 2021). Therefore, we used TGF-β-induced HK-2 cells to further determine whether DSI or Fer-1 treatment had an anti-fibrosis effect *in vitro*. CCK8 assay showed that the concentrations of DSI (0.78, 1.56 and 3.12 μL/mL), which were selected for the following study, did not affect cell proliferation, but could effectively improve the downregulated cell viability induced by TGF-β (Figures 3A,B). In accordance with the results *in vivo*, Figures 3C–F showed that DSI or Fer-1 mitigated the increased expressions of Vimentin and α-SMA, whereas enhanced the decreased expression of E-cadherin *in vivo*. Taken together, these data revealed that DSI or Fer-1 suppressed TGF-β-induced fibrosis *in vitro*.

### DSI attenuated ferroptosis in UUO-induced mice

**FIGURE 4:**
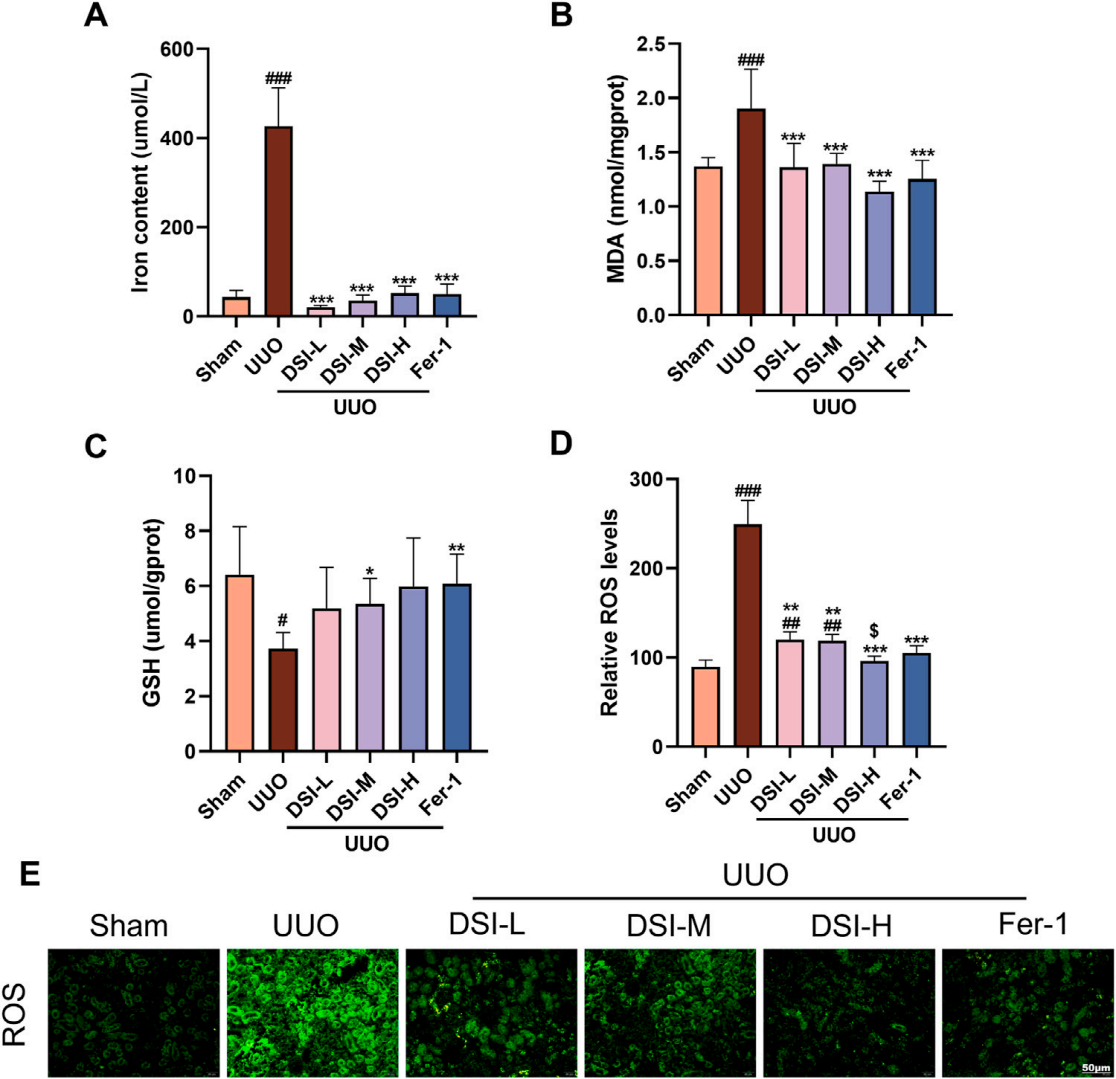
DSI attenuated ferroptosis in UUO-induced mice. **(A–C)** The levels of iron contents, MDA and GSH in kidneys. **(D, E)** The Representative images and quantification of ROS analyzed by immunofluorescence study (50 μm). Data were expressed as the mean ± SD (*n* = 5). ^*#*^ *p* < 0.05 and ^*###*^ *p* < 0.001 vs Sham group, ^*^ *p* < 0.05, ^**^ *p* < 0.01 and ^***^ *p* < 0.001 vs UUO group, ^*$*^ *p* < 0.05 vs 0.78 μL/mL DSI group.

In order to confirm the occurrence of ferroptosis in UUO-induced mice and the effect of DSI on it, the main indicator of ferroptosis, iron content, was first measured. As shown in Figure 4A, iron content was dramatically elevated in the UUO group compared with that in the sham group, which was effectively decreased after DSI intervention (Figure 4A). As GSH, lipid peroxidation and reactive oxygen species (ROS) accumulation are typical signs of ferroptosis, we then determined GSH, lipid-associated product MDA and ROS in kidney tissues. Our findings showed that DSI increased the level of GSH while decreasing MDA level in UUO-induced mice (Figures 4B,C). Immunofluorescence data revealed that DSI weakened the fluorescence intensity of ROS in UUO-induced mice (Figures 4D,E). Collectively, these results proved the notion that ferroptosis existed in UUO-induced mice and this might be a possible mechanism by which DSI exerts antifibrotic properties.

### DSI ameliorated TGF-β-induced ferroptosis in HK-2 cells

**FIGURE 5:**
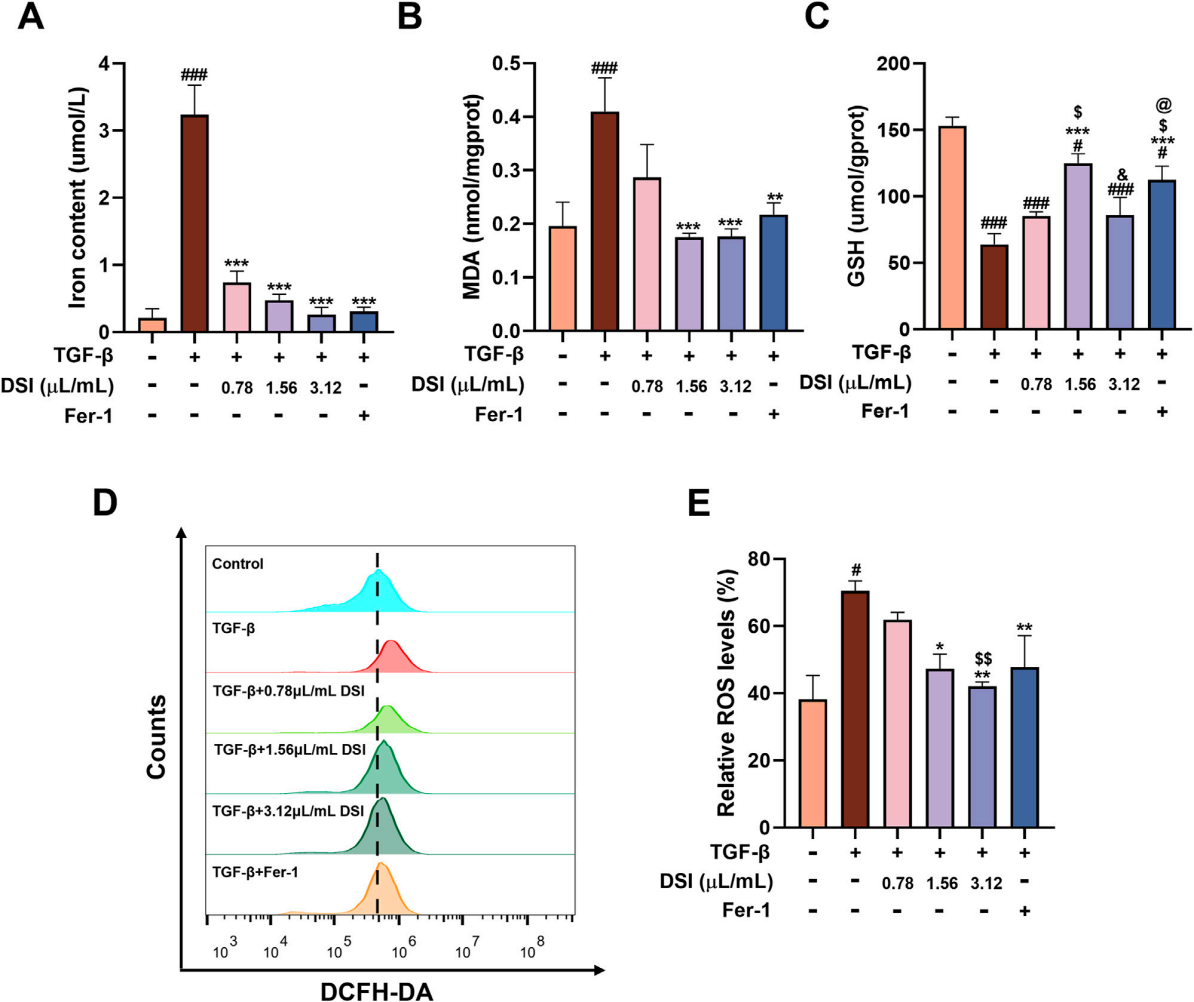
DSI ameliorated TGF-β-induced ferroptosis in HK-2 cells. **(A–C)** The expressions of iron contents, MDA and GSH in HK-2 cells. **(D, E)** The Representative images and quantification of ROS detected by flow cytometry. Data were expressed as the mean ± SD (*n* = 3). ^*#*^ *p* < 0.05 and ^*###*^ *p* < 0.001 vs Control group, ^*^ *p* < 0.05, ^**^ *p* < 0.01 and ^***^ *p* < 0.001 vs TGF-β group, ^*$*^ *p* < 0.05 and ^*$$*^ *p* < 0.01 vs 0.78 μL/mL DSI group, ^*&*^ *p* < 0.05 vs 1.56 μL/mL DSI group, ^*@*^ *p* < 0.05 vs 3.12 μL/mL DSI group.

Next, TGF-β-induced HK-2 cells were used to investigate whether the activity of DSI in reducing renal fibrosis depends on ferroptosis. As shown in Figures 5A–E, compared with the control group, the levels of iron content, as well as MDA and ROS, were abnormally induced in TGF-β-induced HK-2 cells. Besides, the level of GSH was notably suppressed in the TGF-β group. Nevertheless, the aberrant expressions of the abovementioned indicators were significantly attenuated by DSI or Fer-1 administration. These data above revealed that the expressions of ferroptosis-related indicators were greatly altered *in vitro*, which might provide insights into the investigation of DSI regulating ferroptosis against renal fibrosis.

### DSI suppressed ferroptosis stimulated by erastin in HK-2 cells

**FIGURE 6:**
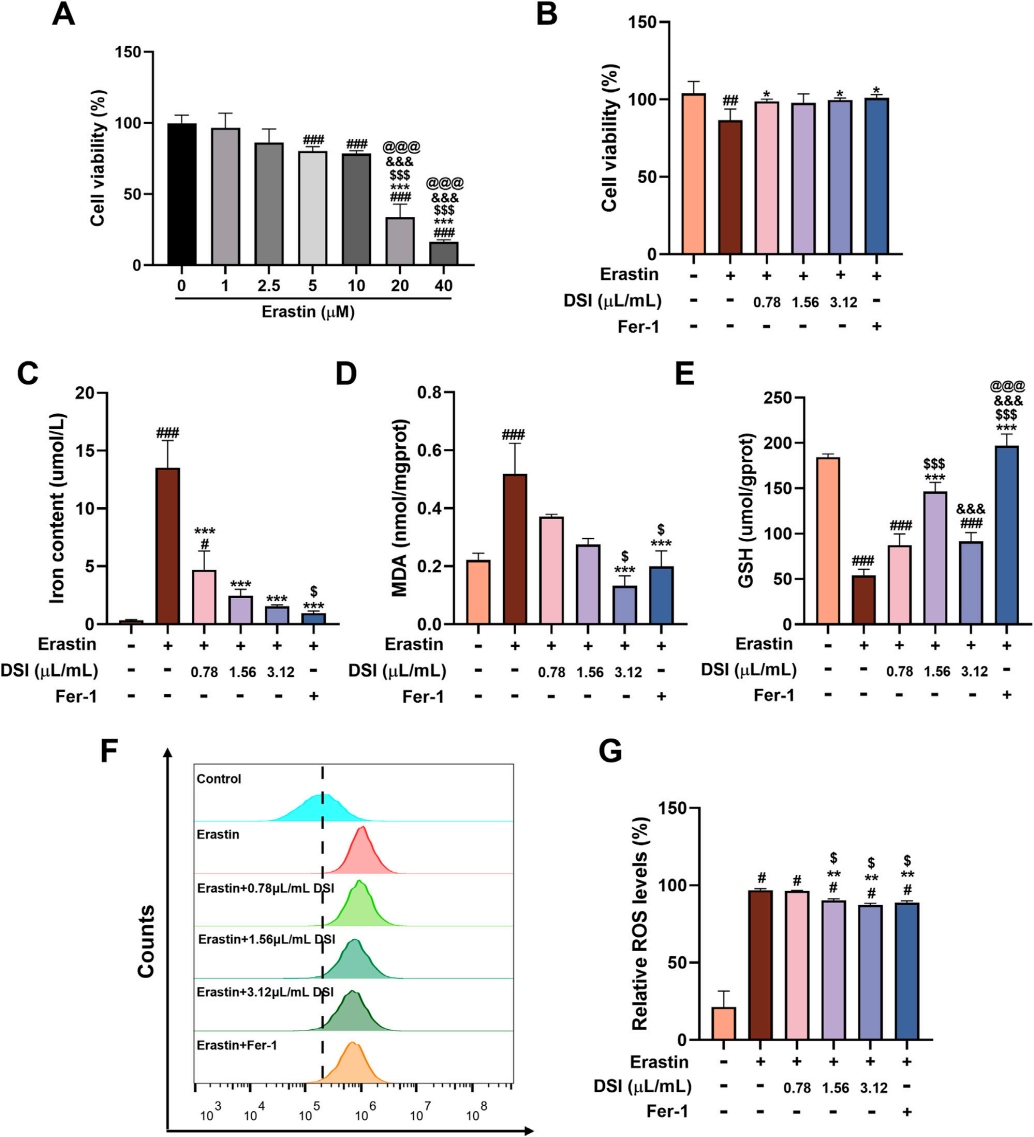
DSI suppressed ferroptosis induced by Erastin in HK-2 cells. **(A–B)** The cell viability of HK-2 cells tested by CCK8 assay kit (^*###*^ *p* < 0.001 vs Control group, ^***^ *p* < 0.001 vs 1 μM Erastin group, ^*$$$*^ *p* < 0.001 vs 2.5 μM Erastin group, ^*&&&*^ *p* < 0.001 vs 5 μM Erastin group, ^*@@@*^ *p* < 0.001 vs 10 μM Erastin group). **(C–E)** The expressions of iron contents, MDA and GSH in HK-2 cells. **(F, G)** The Representative images and quantification of ROS detected by flow cytometry. Data were expressed as the mean ± SD (*n* = 3). ^*#*^ *p* < 0.05 ^*##*^ *p* < 0.01 and ^*###*^ *p* < 0.001 vs Control group, ^*^ *p* < 0.05, ^**^ *p* < 0.01 and ^***^ *p* < 0.001 vs Erastin group, ^*$*^ *p* < 0.05 and ^*$$$*^ *p* < 0.001 vs 0.78 μL/mL DSI group, ^*&&&*^ *p* < 0.001 vs 1.56 μL/mL DSI group, ^*@@@*^ *p* < 0.001 vs 3.12 μL/mL DSI group.

To better elaborate the regulating influence of DSI on ferroptosis, Erastin, a traditional ferroptosis inducer, was employed to trigger ferroptosis damage *in vitro*. CCK8 assay findings manifested that the cell viability decreased along with the elevated concentrations of Erastin (Figure 6A). Intriguingly, DSI abolished cell death caused by Erastin in HK-2 cells (Figure 6B). Consistently, the upregulation of iron content and MDA, as well as the downregulation of GSH induced by Erastin were also mitigated by DSI (Figures 6C–E). Moreover, HK-2 cells incubated with Erastin resulted in an accumulation of ROS, whereas treatment with DSI or Fer-1 terminated this situation (Figures 6F,G).

### Molecular docking between representative components and SIRT1

**FIGURE 7:**
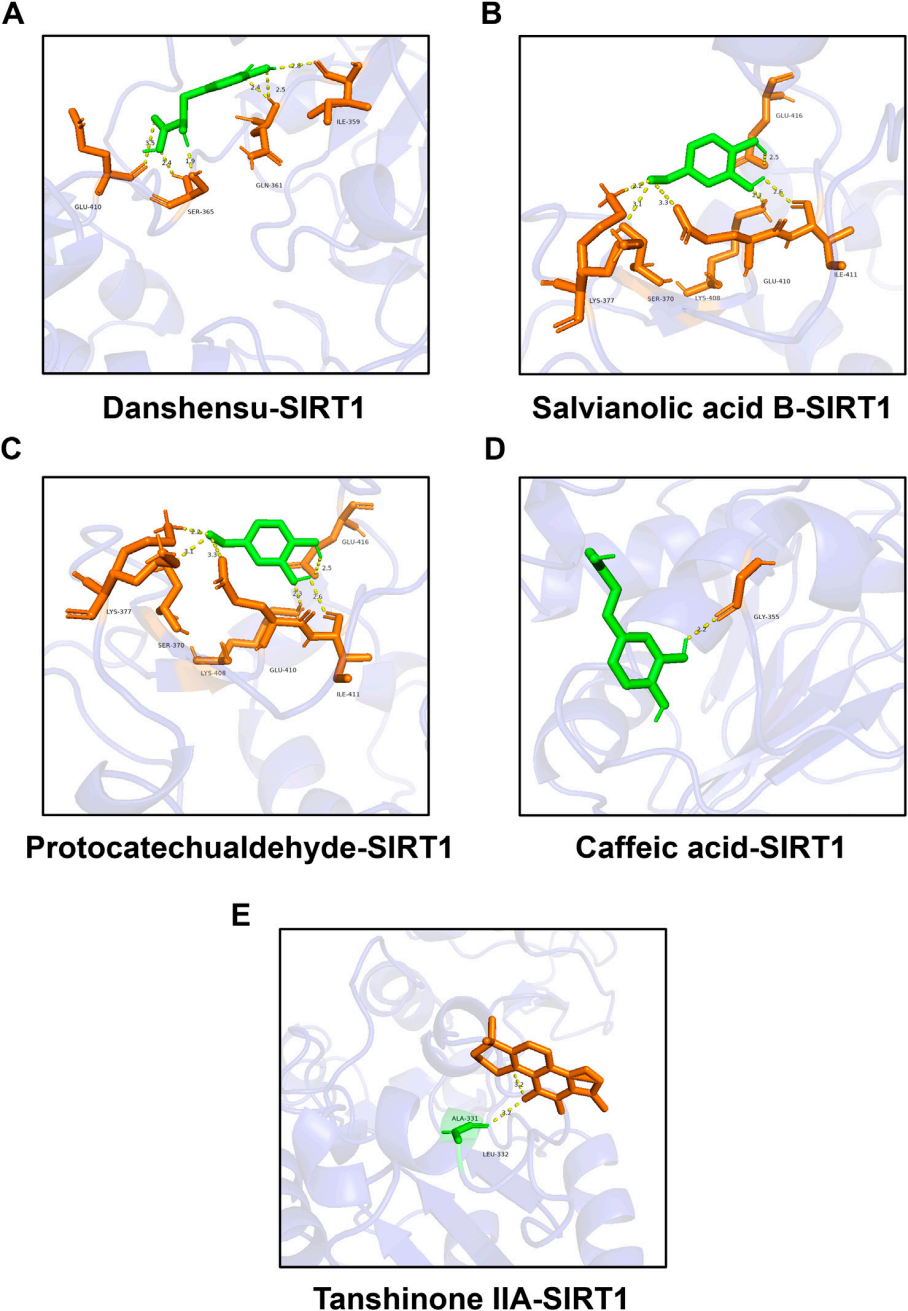
Molecular docking between representative components and SIRT1. **(A)** Molecular docking between danshensu and SIRT1. **(B)** Molecular docking between salvianolic acid B and SIRT1. **(C)** Molecular docking between protocatechuic aldehyde and SIRT1. **(D)** Molecular docking between caffeic acid and SIRT1. **(E)** Molecular docking between tanshinone IIA and SIRT1.

Studies have manifested that the activation of SIRT1 can inhibit ferroptosis (Lv et al., 2024; Tang et al., 2024; Yu et al., 2024). We selected DSI related active ingredients for molecular docking with SIRT1 to predict whether DSI could regulate SIRT1 to exert its effect. The results showed that they both had a good combination with SIRT1. Among them, the binding energies of danshensu, salvianolic acid B, protocatechuic aldehyde, caffeic acid and tanshinone IIA with SIRT1 were −5.8, −8.9, −5.0, −5.8 kcal/mol, respectively (Figures 7A–E). Thus, these results supported the likelihood that SIRT1 had the potential as a target of DSI to inhibit ferroptosis.

### DSI increased the levels of SIRT1 and GPX4 *in vivo* and *in vitro*

**FIGURE 8:**
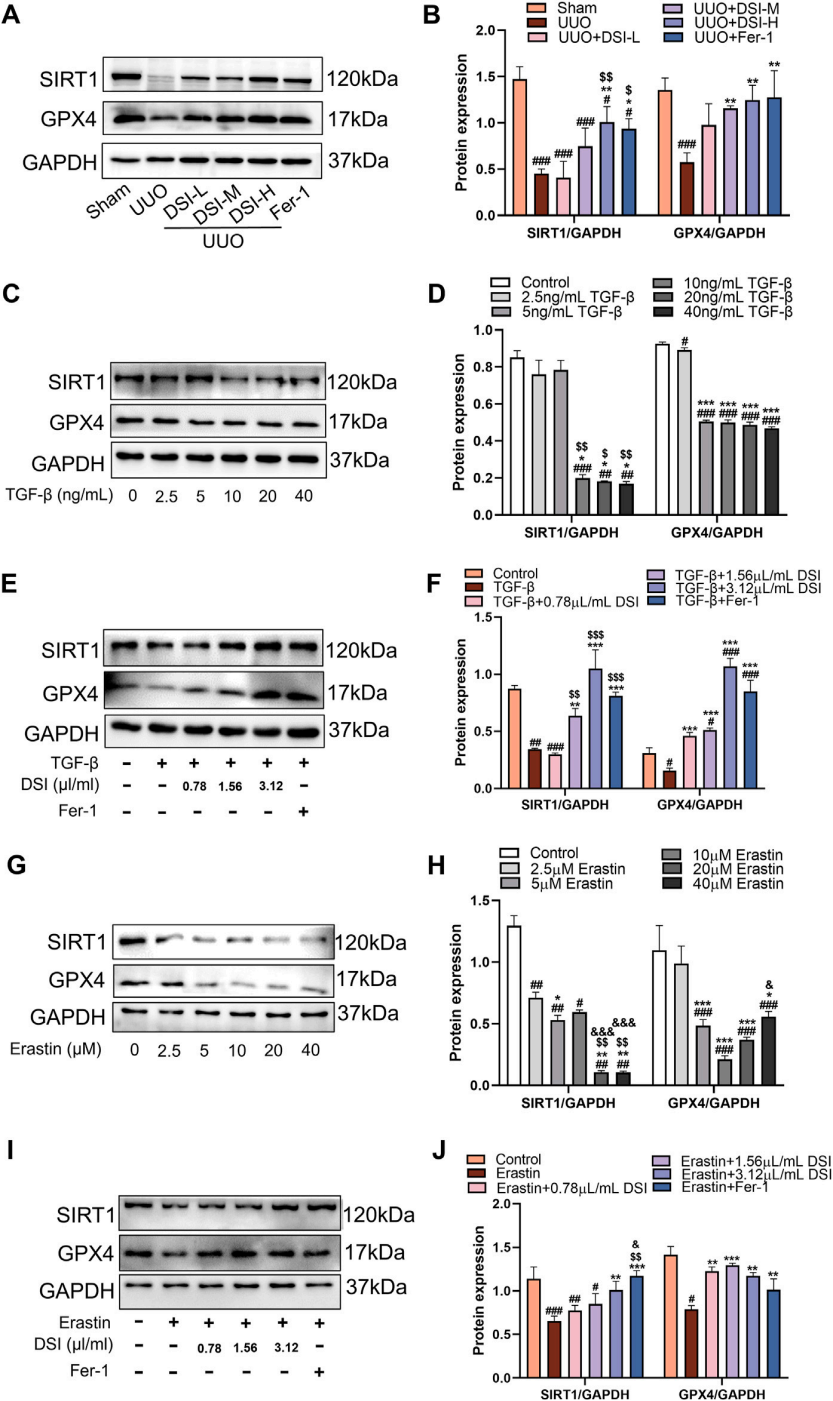
DSI increased the levels of SIRT1 and GPX4 *in vivo* and *in vitro*. **(A–B)** Western blot analyses and quantitative data of SIRT1 and GPX4 in UUO mice. ^*###*^ *p* < 0.001 vs Sham group, ^*^ *p* < 0.05 and ^**^ *p* < 0.01 vs UUO group, ^*$*^ *p* < 0.05 and ^*$$*^ *p* < 0.01 vs DSI-L group. **(C–D)** Western blot analyses and quantitative results of SIRT1 and GPX4 in HK-2 cells. ^*##*^ *p* < 0.01 and ^*###*^ *p* < 0.001 vs Control group, ^*^ *p* < 0.05 and ^***^ *p* < 0.001 vs 2.5 ng/mL TGF-β group, ^*$*^ *p* < 0.05 and ^*$$*^ *p* < 0.01 vs 5 ng/mL TGF-β group. **(E–F)** Western blot analyses and quantitative results of SIRT1 and GPX4 in HK-2 cells. ^*#*^ *p* < 0.05, ^*##*^ *p* < 0.01 and ^*###*^ *p* < 0.001 vs Control group, ^**^ *p* < 0.01 and ^***^ *p* < 0.001 vs TGF-β group, ^*$$*^ *p* < 0.01 and ^*$$$*^ *p* < 0.001 vs 0.78 μL/mL DSI group. **(G–H)** Western blot analyses and quantitative results of SIRT1 and GPX4 in HK-2 cells. ^*##*^ *p* < 0.01 and ^*###*^ *p* < 0.001 vs Control group, ^*^ *p* < 0.05, ^**^ *p* < 0.01 and ^***^ *p* < 0.001 vs 2.5 μM Erastin group, ^*$$*^ *p* < 0.01 vs 5 μM Erastin group, ^*&*^ *p* < 0.05 and ^*&&&*^ *p* < 0.001 vs 10 μM Erastin group. **(I–J)** Western blot analyses and quantitative results of SIRT1 and GPX4 in HK-2 cells. ^*#*^ *p* < 0.05 ^*##*^ *p* < 0.01 and ^*###*^ *p* < 0.001 vs Control group, ^**^ *p* < 0.01 and ^***^ *p* < 0.001 vs TGF-β group, ^*$$*^ *p* < 0.01 vs 0.78 μL/mL DSI group, ^*&*^ *p* < 0.05 vs 1.56 μL/mL DSI group. Data were expressed as the mean ± SD (*n* = 3).

GPX4 as a central regulator, modulates ferroptosis by reducing ROS accumulation and curbing cellular iron uptake (Ni et al., 2023). We used Western blot to investigate the involvement of SIRT1 and GPX4 in the progression of renal fibrosis. Compared to the UUO group, the levels of SIRT1 and GPX4 were increased in the DSI or Fer-1 treatment group (Figures 8A,B). In HK-2 cells, the protein levels of SIRT1 and GPX4 were gradually decreased with the concentrations of TGF-β (Figures 8C,D) or Erastin increased (Figures 8G,H), which means that inducing ferroptosis could inhibit the levels of SIRT1 and GPX4. Interestingly, the levels of SIRT1 and GPX4 were increased in different degrees by DSI or Fer-1 intervention under TGF-β (Figures 8E,F) or Erastin stimulation (Figures 8I,J). In total, these findings suggested that the reduction of renal fibrosis by DSI treatment may be due to the activation of SIRT1 and GPX4.

### SIRT1 inhibition reversed the effects of DSI on ferroptosis and renal fibrosis

**FIGURE 9:**
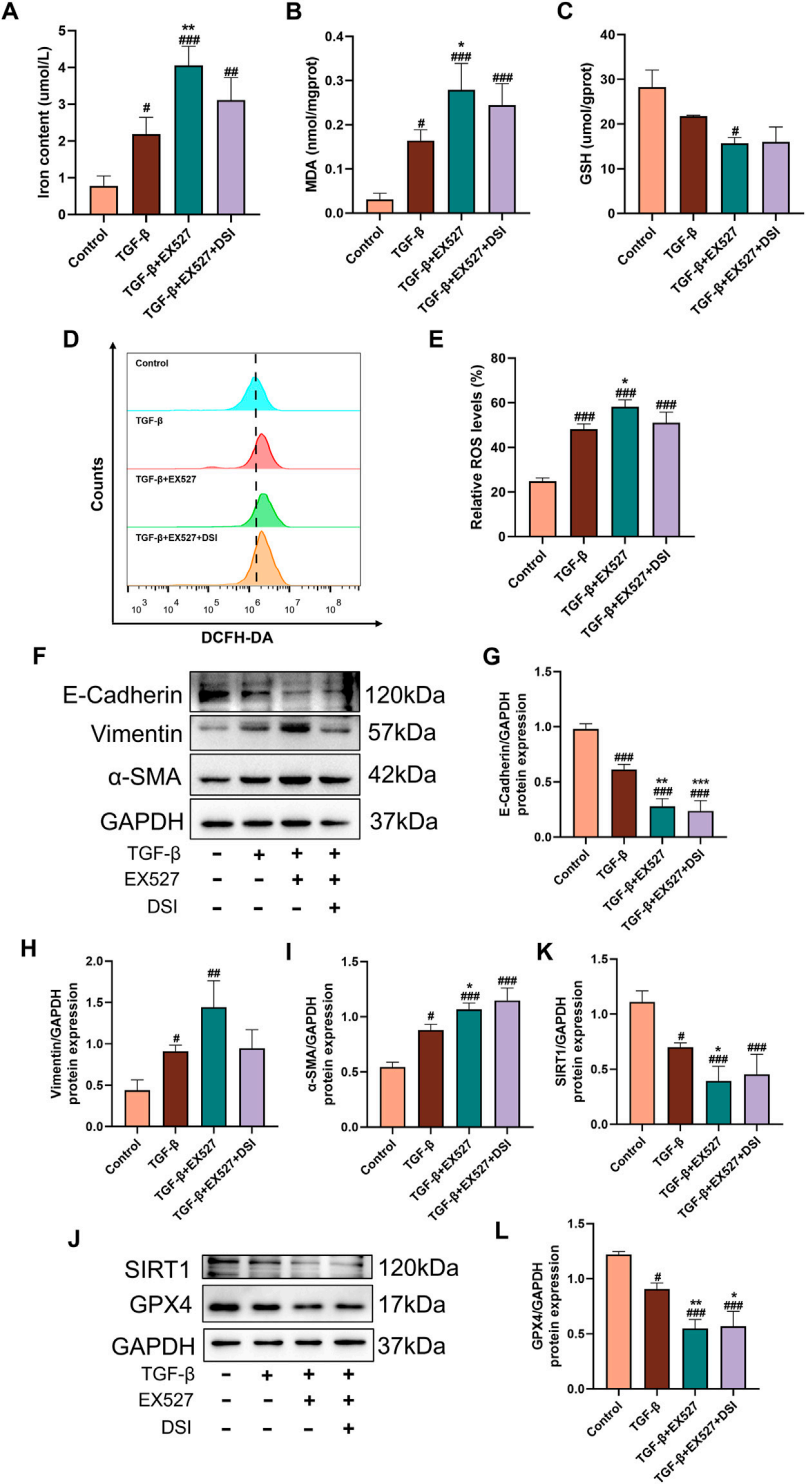
SIRT1 inhibition blocked the effects of DSI on ferroptosis and renal fibrosis. **(A–C)** The expressions of iron contents, MDA and GSH in HK-2 cells. **(D, E)** The Representative images and quantification of ROS detected by flow cytometry. **(F–L)** Western blot analyses and quantitative results of E-Cadherin, Vimentin, α-SMA, SIRT1 and GPX4. Data were expressed as the mean ± SD (*n* = 3). ^*#*^ *p* < 0.05 ^*##*^ *p* < 0.01 and ^*###*^ *p* < 0.001 vs Control group, ^*^ *p* < 0.05, ^**^ *p* < 0.01 and ^***^ *p* < 0.001 vs TGF-β group.

To further study the role of SIRT1 in ferroptosis and renal fibrosis, SIRT1 inhibitor EX527 was utilized to inhibit the activation of SIRT1 in HK-2 cells. The results verified that compared to the TGF-β group, EX527 aggravated ferroptosis related indicators and the expressions of EMT-related proteins induced by TGF-β, as well as decreased the expressions of SIRT1 and GPX4 (Figures 9A–L), which abrogated the protective effects of DSI on ferroptosis and renal fibrosis.

## Discussion

“Homotherapy for heteropathy” is the basic principle of treating diseases in traditional Chinese medicine (TCM), which means that different diseases with similar pathogenesis can adopt the same treatment method during their onset (Liao et al., 2024). DSI is a representative TCM for activating blood and eliminating stasis, which is widely used to treat cardiovascular diseases (CVDs) in clinic, including angina and heart failure (Li et al., 2022b; Song et al., 2024). Recently, DSI and its active components have been reported to possess a nephroprotective effect in acute kidney injury (AKI) and CKD (Sun et al., 2022; Xu et al., 2024). TCM believes that the pathogenesis of CVDs and CKD are both deficiency in essence and excess in substance. One of the strategies of TCM in treating them is to promote blood circulation and remove blood stasis (Wang et al., 2022). Renal fibrosis is a typical pathological alteration in the pathogenesis of CKD. Given the powerful blood activating and stasis removing functions of DSI, it has great potential in the treatment of renal fibrosis. In this study, we discovered that DSI showed renoprotective benefits against UUO-induced renal function deterioration, with improvements in morphological and pathological changes (Figure 1).

DSI contains many active ingredients, including danshensu, salvianolic acid A, salvianolic acid B, salvianolic acid C, protocatechuic aldehyde, caffeic acid and tanshinone IIA, which exert various pharmacological activities such as anti-inflammatory, anti-apoptosis, anti-oxidative stress and anti-fibrosis to protect renal diseases (Chien et al., 2021; Fan et al., 2024; Chen et al., 2022). Emerging evidence revealed that DSI and its active components alleviated many diseases like myocardial ischemia reperfusion injury, myocardial infarction and ischemic brain injury via regulating ferroptosis (Ko et al., 2023; Shen et al., 2022; Hu et al., 2023; Wu et al., 2024). Ferroptosis is a type of iron-dependent programmed cell death, which is different from apoptosis, cell pyroptosis, cell necrosis and autophagy, closely associated with the pathological process of many diseases (Van Coillie et al., 2022). Multiple studies have demonstrated that ferroptosis participates in the process of renal fibrosis, whereas inhibiting ferroptosis could mitigate kidney injury (Zhang et al., 2022). In this regard, it is worthwhile to assess the adaptable role of DSI against renal ferroptosis. Notably, DSI alleviated the increased iron, the decreased GSH, and the increased lipid peroxide MDA in tubular cells ferroptosis triggered by Erastin (Figure 6). In addition, these phenomena were also validated in the kidneys of renal fibrosis mice and in tubular cells stimulated by TGF-β, respectively (Figures 4, 5). Although Fer-1 didn’t affect the decline of cell viability induced by TGF-β (Figure 3B), which has been confirmed in a reported literature (Kim et al., 2021), Fer-1 still exerts marked effects on attenuating ferroptosis *in vivo* and *in vitro*, which further corroborates that ferroptosis serves as a critical role during renal fibrosis. Contrary to the results *in vivo*, Fer-1 could not prevent the decreased of E-Cadherin caused by TGF-β (Figures 3C,D). Since the regulatory effect of Fer-1 on EMT varies with different pathogenic factors and cell types (Liu et al., 2023; Xie et al., 2022), more researches are required to investigate the role of Fer-1 in EMT. Taken together, these results demonstrated that DSI suppressed ferroptosis in the progression of renal fibrosis.

SIRT1, a nicotinamide adenine dinucleotide (NAD+)-dependent histone deacetylase, is the most widely studied members of sirtuins in AKI and CKD, which exerts protective effects by regulating cell apoptosis, oxidative stress, inflammation, autophagy, ferroptosis and fibrosis (Perico et al., 2024; Qiongyue et al., 2022). A recent study has illustrated that salvianolic acid C could activate SIRT1 to protect the kidney from injury (Chien et al., 2021), which inspired us that DSI may have a regulatory effect on SIRT1 to exert renoprotective effect. Our molecular docking results confirmed our hypothesis (Figure 7). Evidence proves that stabilizing SIRT1 expression can inhibit ferroptosis (Ma et al., 2020). GPX4, the critical regulator of ferroptosis, utilizes GSH as a cofactor to remove lipid peroxides and restrict the iron-dependent accumulation of ROS, hence preventing ferroptosis (Forcina and Dixon, 2019). Our study found that the expressions of SIRT1 and GPX4 were continuously decreased by the elevated concentrations of TGF-β or Erastin in tubular cells and in renal fibrosis model mice, which were blocked by Fer-1 (Figure 8), indicating that the downregulations of SIRT1 and GPX4 were associated with the trigger of ferroptosis in renal fibrosis. Specifically, SIRT1 deacetylates FOXO3A to activate GPX4 transcription, alleviating cisplatin-induced lipid peroxidation and ROS accumulation, whereas inhibition of SIRT1 reduced GPX4 expression, aggravating renal ferroptosis (Fang et al., 2021; Qiu et al., 2024). Consistent with the reported literature, renal ferroptosis and EMT process induced by TGF-β were aggravated when the activation of SIRT1 was inhibited by EX527, causing the effect of DSI was weakened or even disappeared (Figure 9). Taken together, our data suggested that DSI protected renal fibrosis against ferroptosis may be partially via SIRT1/GPX4 activation, highlighting that the potential effects of DSI as a novel therapeutic drug for limiting renal fibrosis.

To be honest, this study had some limitations that could be addressed in future. First, although DSI was shown to suppress ferroptosis in our study, this finding is the integrated effect of many components of DSI. Further studies into the exact bioactive ingredient of DSI are required to enhance an understanding of its mechanism of action. In addition, further researches are still needed to determine the exact mechanism of SIRT1 on GPX4 in renal fibrosis.

## Conclusion

In conclusion, our research illustrated that DSI could attenuate renal fibrosis *in vivo* and *in vitro* by reducing ferroptosis for the first time. The protective effects of DSI were mediated through SIRT1/GPX4 pathway. Thus, DSI could be regarded as a hopeful candidate drug for delaying the process of renal fibrosis and its potential mechanism is worthy of further investigation.

## Data Availability

The raw data supporting the conclusions of this article will be made available by the authors, without undue reservation.

## Abbreviations

BUN, Blood urea nitrogen; CKD, Chronic kidney disease; DSI, Danshen Injection; ECM, Extracellular matrix; EMT, Epithelial-mesenchymal transition; Fer-1, Ferrostatin-1; GPX4, Glutathione peroxidase 4; GSH, Glutathione; MDA, Malonaldehyde; ROS, reactive oxygen species; SCr, Serum creatinine; SIRT1, Sirtuin-1; TGF-β, Transforming growth factor-β; UUO, Unilateral ureteral obstruction; α-SMA, α-smooth muscle actin.
